# Performance of a new natural oral contrast agent (LumiVision®) in dynamic MR swallowing

**DOI:** 10.1007/s00330-021-07927-5

**Published:** 2021-04-24

**Authors:** Christiane Kulinna-Cosentini, Michael A. Arnoldner, Wolfgang Schima, Ivan Kristo, Sebastian F. Schoppmann, Michael Weber, Enrico P. Cosentini

**Affiliations:** 1 Department of Biomedical Imaging and Image-guided Therapy, Medical University of Vienna, Waehringer Guertel 18-20, 1090 Vienna, Austria; 2 Department of Diagnostic and Interventional Radiology, Göttlicher Heiland Krankenhaus, Barmherzige Schwestern Krankenhaus, and Sankt Josef Krankenhaus, Vienna, Austria; 3 Department of General Surgery, Medical University of Vienna, Vienna, Austria

**Keywords:** Esophagus, Magnetic resonance imaging, Fundoplication, contrast agent

## Abstract

**Objectives:**

To evaluate image quality by first use of LumiVision® in dynamic MR swallowing, a contrast medium consisting of biological substances versus a gadolinium-buttermilk mixture in patients who underwent Nissen fundoplication due to gastroesophageal reflux disease (GERD).

**Methods:**

The protocol of this retrospective study was approved by the local Institutional Review Board. A hundred twenty-nine patients (146 examinations) underwent a dynamic MR swallowing study (at 1.5 T or 3.0 T) and received an oral contrast agent. Two readers evaluated the distention of the esophagus, contrast, and traceability of the bolus in a 3-point scale. A steady-state coherent sequence (B-FFE, TrueFISP) was used. The patients were divided into 3 different groups: 53 patients received gadolinium chelate (Dotarem®)–buttermilk mixture (GBM) in a dilution of 1:40 as an oral contrast agent; 44 patients received LumiVision® water mixture (LWM) in a dilution of 1:1 and 49 patients received LumiVision® (L) undiluted.

**Results:**

GBM showed significantly better results in overall evaluation for both readers in contrast to LWM (*p* = .003, *p* = .002). L also reached significantly better results in overall evaluation than LWM in both readers (*p* = .004, *p* = .042). There was no significant difference in the overall evaluation between L and GBM (*p* = .914, *p* = .376).According to Landis and Koch, interobserver agreement was “substantial” (Cohen’s kappa = 0.738) between both readers.

**Conclusion:**

LumiVision® undiluted showed equal image quality compared to gadolinium-buttermilk mixture. The constellation of LumiVision® water mixture led to a clearly negative result in relation to the image quality compared to LumiVision® undiluted. Therefore, oral ingestion of LumiVision® undiluted is recommended for MR swallowing examinations.

**Key Points:**

*• LumiVision® undiluted shows significantly better image quality in comparison to LumiVision® diluted in oral application in swallowing MRI.*

*• LumiVision® undiluted shows equal image quality in comparison to gadolinium-buttermilk mixture in oral application.*

*• Oral ingestion of LumiVision® undiluted can replace gadolinium-buttermilk mixture in oral MR examinations*.

## Introduction

Dynamic magnetic resonance imaging (MRI) is a new imaging method for the evaluation of the esophagus since technical advances have improved spatial and temporal resolution with reduction of artifacts [1–4]. The basic requirements for achieving high image quality in dynamic MR swallowing examinations are complete distention of the esophagus and good contrast between the esophagus and surrounding soft tissue structures as well as appropriate traceability of the oral contrast agent bolus. Recent improvements in MR imaging of the gastroesophageal tract allow detection of wrap dysfunction, alterations of esophageal peristalsis, and motility [5, 6] of symptomatic patients after Nissen fundoplication due to GERD. Several studies have dealt with this topic, but no unique recommendation concerning the ideal oral contrast agent has been reached [2–5]. Several studies considered different contrast agents to enhance the signal of the digestive tract [7–9]. Other studies dealt with natural contrast agents like pineapple or blueberry juice [6, 10]. Another group used fluids or thickened fluids like buttermilk spiked with gadolinium chelate contrast agents [3, 5]. In times of public discussions about the toxicity of gadolinium contrast agents, natural substances of fruit juices, which we are used to drink or eat often, may be preferred. So the purpose of this study was to evaluate different oral contrast agents in MR swallowing studies in symptomatic patients after Nissen fundoplication, and to examine if the buttermilk-gadolinium mixture could be replaced by LumiVision®. LumiVision® is a semiliquid preparation of concentrates from pineapple, organic agave syrup, blackcurrant, guar gum, and defoamers. It contains no preservatives and no dyes. The feasibility of esophagus visualization with oral administration of a recently developed oral contrast agent LumiVision®, (Bender group) in dilution and undiluted, was compared to the well-known buttermilk-gadolinium mixture [3–5] during MRI.

## Methods

### Patients

Ethical approval for this retrospective study of available clinical data was obtained by the Institutional Review Board. An informed consent was waived by the ethics committee.

Between June 2014 and February 2018, 129 patients (61 male, 68 female; mean age, 59 years; range, 31–73 years) with persistent, recurrent, or new symptoms were enrolled in the study. Leading symptoms included heartburn, regurgitation, and dysphagia. Five patients underwent more than one MR examination, so 146 MR examinations were evaluated overall. All patients had undergone laparoscopic antireflux surgery with Nissen fundoplication and presented with new or recurrent symptoms after operation. The symptoms recurred at 2–30 months after operation and had existed for 4–35 months at the time of MRI.

The contrast medium and the dose varied with the time. Until 2015, we used gadolinium-buttermilk mixture, because LumiVision was not available at that time. From 2015 onwards, we then first worked with diluted LumiVision, as it was recommended by the Bender group, followed by application of undiluted LumiVision.

### MR fluoroscopy

MR imaging was performed either on a 3.0 T (Magnetom Trio 3T, Siemens Medical Solutions) or on a 1.5 T MRI scanner (Intera, Philips) with a phased array coil placed upon the chest. In addition to clinical inclusion criteria, general inclusion criteria comprised no contraindications for MRI and age over 18. Pregnant patients and patients who were unable to swallow in a supine position were excluded from our study. Prior to the MR exam, the clinical history was obtained by one of the participating radiologists, and the patient’s ability to swallow in the supine position was tested.

The following image protocol was performed to locate the esophagus and its course, then to locate the GEJ with the exact position of the wrap, and last to evaluate the functionality of esophageal motility: after a reference scan, a coronal T2-weighted half-Fourier acquired single-shot turbo spin-echo (HASTE) sequence was obtained to depict the complete course of the esophagus, anatomic landmarks like upper esophageal sphincter region, and gastroesophageal junction in three orientations.

To determine the optimum slice angle, a sagittal oblique steady free precession sequence (SSFP)**,** either B-FFE (balanced fast-field echo sequence, on 1.5 T) or TrueFISP (true fast imaging with steady-state precession, on 3.0 T MRI), was centered on the esophagus, as seen on the coronal T2w HASTE sequence. The sagittal oblique SSFP sequence was performed as a pulse sequence with three contiguous slices for better coverage of the entire course of the esophagus and to compensate if the esophagus slipped out of the imaging plane due to breathing. Pulse sequences were repeated, if a delay of esophageal emptying was observed in the first dynamic sequence. The sequence parameters are listed in Table 1.

**Table 1:** MR sequence parameters

Parameters	HASTE	B-FFE	TrueFisp
Repetition time, TR (ms)	1800	2.9	2.04
Echo time, TE (ms)	100	1.5	0.82
Flip angle (°)	150	60	8
Acquisition matrix	256 × 256	256 × 256	256 × 256
Field of view, FOV (mm)	350 × 350	375 × 375	
Slice thickness (mm)	5	15	15
Intersection gap (mm)	-	0.4	-
Aquisition time (s/image)	1	1	1
Acquisition cycle (s)	-	60	60
Slice orientation	1. Coronal	1. Sagittal oblique	1. Sagittal oblique
	2. Sagittal	2. Coronal oblique	2. Coronal oblique
	3. Axial		

The oral contrast medium was placed in a cup with a long plastic tube and was placed near the patient’s head in the MR gantry, so that the other end of the plastic tube could be placed into the patient’s mouth. Patients were instructed to take a bolus of the oral contrast agent and to swallow it in a single gulp, then open their mouth after each swallow to prevent repetitive swallowing. If coverage of the esophagus was inadequate, the pulse sequence was repeated at a slightly different angulation. Three single boluses were scanned in that way.

The patients were divided into three groups depending on the oral contrast medium: the first patient group received the buttermilk-gadolinium mixture (gadoterate meglumine, Guerbet) (GBM) in a well-established dilution of 40:1 (240-ml buttermilk and 6 ml of gadoterate meglumine) from previous publications [3, 5]. The second group received LumiVision® (Bender group) with water in a dilution of 1:1, as it is recommended from the manufacturer Bender group and has been published [4].

The third group of patients swallowed LumiVision® undiluted. The maximum amount if swallowed LumiVision® did not exceed 250 ml. Choice of given oral contrast agent was made in relation to examination time period. Patients who were examined in 2014–2015 received the gadolinium-buttermilk mixture, patients from 2015 to 2016 received LumiVision® diluted, and patients after 2016 received LumiVision® undiluted. No additional IV contrast medium was administered.

### Image analysis

MR images were independently analyzed by two radiologists (reader 1 with 20 years of experience in abdominal radiology and reader 2 with 8 years of experience) in cine mode and in a frame-by-frame analysis in view of image quality on a PACS Workstation (IMPAX, Agfa-Gevaert). MR images were assessed in random successive order by each radiologist, which were blinded to each other. Each of the 3 MR parameters was evaluated in a separate session to avoid interactions.

For qualitative evaluation of image quality, a three-point scale (1-bad, 2-fair, 3-excellent) was used to score three different parameters:

1. Distention of the esophagus
2. Contrast between esophageal lumen and surrounding structures
3. Traceability of swallowed bolus

For overall evaluation, the sum of average points of the three parameters of both readers has been calculated:

- 3–5 points = bad
- 6–7 points = fair
- 8–9 points = excellent

All evaluations were performed by choosing the best sequences of coronal and sagittal SSFP in cine mode of each examination. Both readers were blinded to the rating results of the other reader.

### Statistical analysis

All statistical analyses were performed using IBM SPSS Statistics for Windows Version 25.0 (IBM Corporation). All data are shown as relative frequencies and percentages. Kruskal-Wallis test and chi-squared test were used to calculate the differences between the three contrast agent groups in the three different image quality parameters. In case of significant differences, additional Bonferroni-corrected Mann-Whitney *U* tests were used as post hoc tests. A value of *p* < .05 was considered the threshold for statistical significance.

In addition to descriptive statistics, interrater reliability for both readers was calculated using Cohen’s kappa according to Landis and Koch [11].

## Results

The present study includes a total of 129 patients (68 female; 61 male), of whom 15 had two and one patient has three examinations. So totally, 146 examinations were performed. All patients received an oral contrast agent. In 53 examinations, patients obtained GBM in a dilution of 1:40, in 44 examinations, the patients received LWM in a dilution of 1:1, and in 49 examinations, patients received L. All patients, who received GBM and LWM as well as 17 patients of the L group, were examined on a 3 T MRI, whereas 32 patients of the L group were examined on a 1.5 T MRI. The mean age at first presentation was 51.9 years (range, 23–79 years).

### Overall rating

The most excellent results in the overall rating were found in the L group with 78% (38/49) in both readers, whereas the GBM group in R1 and R2 with 75% (40/53) and 77% (41/53) showed nearly equal results. The excellent results in the LWM group were significantly worse in R1 and R2 with 50% (22/44). The worst results were found in the LWM group with 27% (12/44) and 23% (10/44) for R1 and R2, whereas only 6% (3/53) was found in the GBM group for both readers and 6% (3/49) and 4% (2/49) were shown in R1 and R2 in the L group (Fig. 3).

The post hoc test showed a statistically significant difference between LWM and GBM (*p* = 0.003), as well as between LWM and L (*p* = .0002) in rating the image quality for reader 1. Between GBM and L, no statistically significant difference (*p* = 0.914) could be evaluated in the total rating for reader 1.

The overall rating in reader 2 showed also a statistically significant difference between LWM and GBM (*p* = 0.001), as well as between LWM and L (*p* = 0.0019). Between GBM and L, no statistically significant difference (*p* = 0.376) could be evaluated. The overall ratings were nearly equal in both readers with regard to the three different groups with an interrater agreement of kappa = 0.738 as “substantial agreement.”

### Distention of the esophagus

Readers 1 and 2 rated distention of the esophagus as bad in 15/146 (10%) and 12/146 (8%), fair in 51/146 (34%) and 48/146 (33%), and excellent in 80/146 (54%) and 86/146 (59%), respectively (Table 2). The highest proportion of bad ratings were reached in the LumiVision® water mixture (LWM) group with 18% (8/44) and 14% (6/44) in both readers (R1 and R2) versus 9% (5/53) and 4% (2/53) in the GBM group and 4% (2/49) and 8% (4/49) in the L undiluted group, respectively.

**Table 2:** Evaluation of esophageal distention

Distention	Contrast agents			
	LWM	GBM	L	Total
Reader 1				
Excellent (patients, %)	18 (41%)	34 (64%)^a^	28 (57%)	80 (55%)
Fair (patients, %)	18 (41%)	14 (27%)	19 (39%)	51 (35%)
Bad (patients, %)	8 (18%)	5 (9%)	2 (4%)	15 (10%)
Reader 2				
Excellent (patients, %)	21 (48%)	36 (68%)^b^	29 (59%)	86 (59%)
Fair (patients, %)	17 (39%)	15 (28%)	16 (33%)	48 (33%)
Bad (patients, %)	6 (14%)	2 (4%)	4 (8%)	12 (8%)
Total (patients)	44	53	49	146

LWM LumiVision® water mixture, GBM gadolinium water mixture

L LumiVision® undiluted

^a^*p* value is 0.04 for GBM versus LWM in reader 1

^b^*p* value is 0.074 for GBM versus LWM in reader 2

**Fig. 1:**
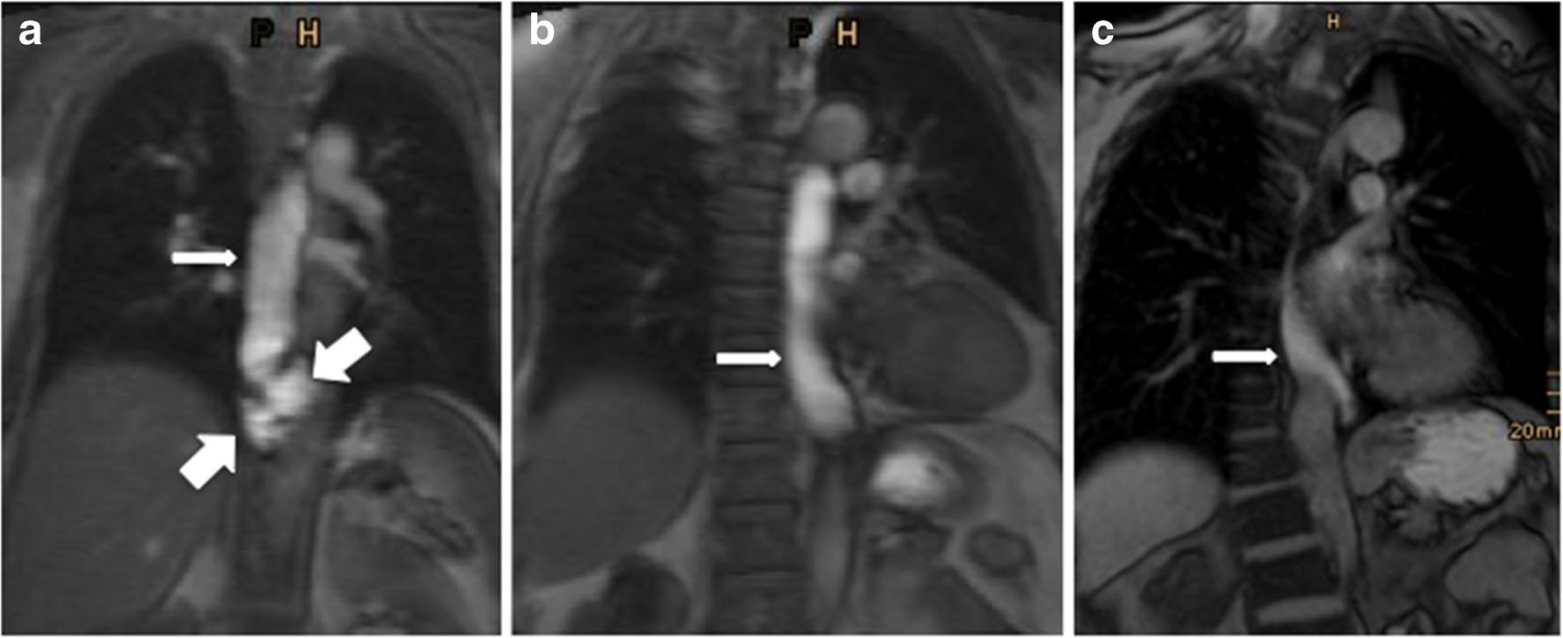
Postoperative appearance after Nissen fundoplication on MRI in three different patients. Contrast medium passes through the esophagus (arrows) and shows excellent distention of the esophagus with gadolinium-buttermilk mixture on a 3T MRI (**a**), as well as with LumiVision® undiluted (**b**) in the coronal view on a 1.5 T MRI. Poor distention is seen with LumiVision® water mixture on a 1.5 T MRI (**c**). In the first patient (**a**), a telescope phenomenon could be seen (big arrows). Parts of the stomach have been slipped through the intact wrap into the thorax

Most excellent distentions were reached in the gadolinium-buttermilk mixture (GBM) group with 64% (34/53) and 68% (36/53) (R1 and R2). The ratings for L undiluted were nearly equal to GBM with 57% (28/49) and 59% (29/49) (R1 and R2), whereas LWM reached 41% (18/44) in R1 and 48% (21/44) in R2 (Fig. 1).

The rating for distention of the esophagus in readers 1 and 2 showed also a statistically significant difference between GBM and LWM (*p* = 0.04/*p* = 0.074) in excellent rating. There was also a clear trend between LWM and L (*p* = 0.183/*p* = 0.4), but no significant difference. Between GBM and L, no statistically significant difference (*p* > 0.5) could be evaluated in both readers.

### Contrast

**Fig. 2:**
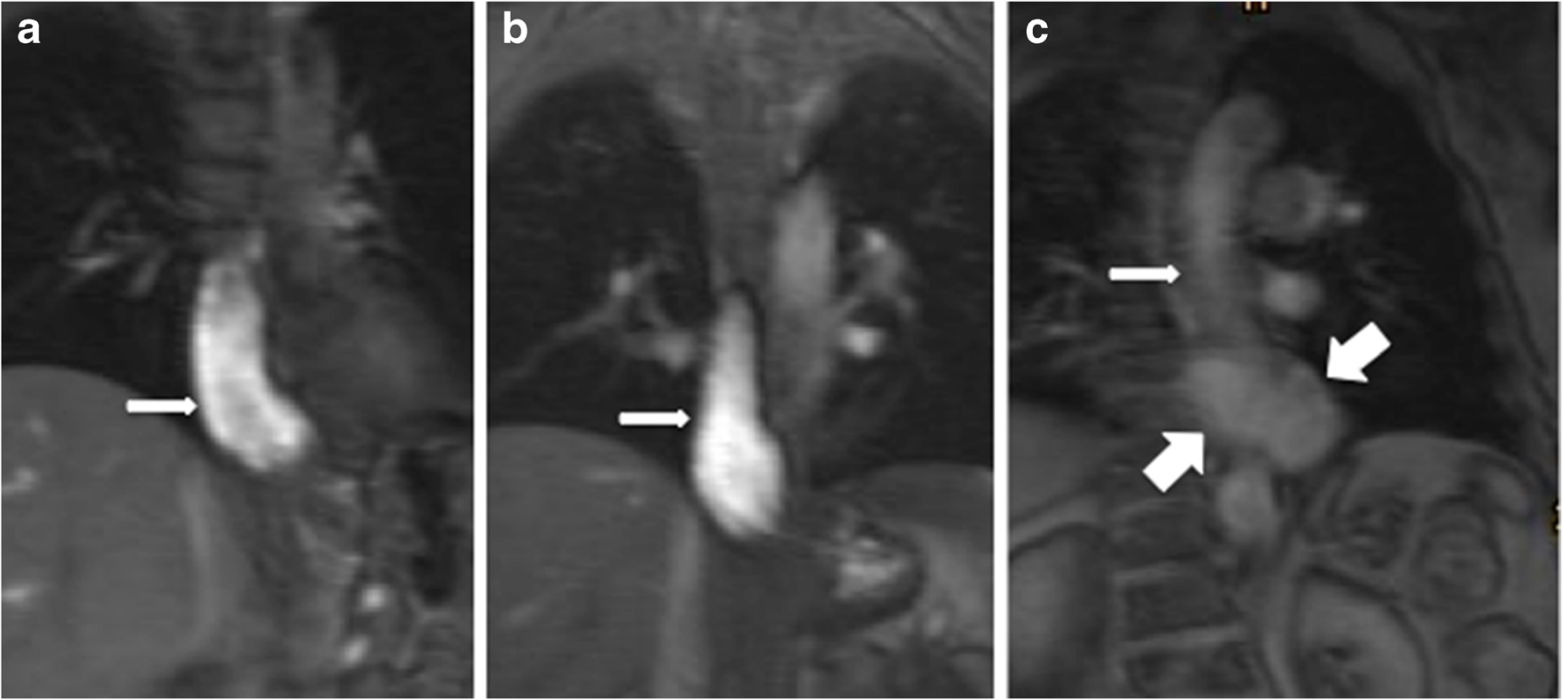
Contrast of the different contrast media in three different patients. TrueFisp sequences in the coronal view were performed to demonstrate the esophagus. All examinations were performed on a 3T MRI. An excellent contrast of the esophagus to the surrounding structures was reached with gadolinium-buttermilk mixture (**a**) as well as with LumiVision® undiluted (**b**). The patient with swallowed LumiVision® water mixture shows a bad contrast of the esophagus (**c**). In this patient, the wrap was ruptured and a big part of the stomach was migrated into the thorax, which is called Re-Hernia (big arrows)

The best contrast of the esophagus versus the surrounding structures was reached by the LumiVision® undiluted (L) group by R1 and R2 in 86% (42/49) and 88% (43/49) with a narrow margin to the GBM group in 83% (44/53) and 85% (45/53). The contrast in the LWM group was rated inferior by R1 and R2, with only 52% (23/44) and 55% (24/44) in the excellent group (Table 3). There was no study, which was rated bad in the GBM and L undiluted group, whereas 16% (7/44) and 13% (6/44) in the LWM group were found to have bad contrast (Fig. 2).

**Table 3:** Evaluation of contrast

Contrast	Contrast agents			
	LWM	GBM	L	Total
Reader 1				
Excellent (patients, %)	23 (52%)	44 (83%)^a^	42 (86%)^b^	109 (75%)
Fair (patients, %)	14 (32%)	9 (17%)	7 (14%)	30 (20%)
Bad (patients, %)	7 (16%)	0 (0%)	0 (0%)	7 (5%)
Reader 2				
Excellent (patients, %)	24 (55%)	45 (85%)^c^	43 (88%)^d^	112 (77%)
Fair (patients, %)	14 (32%)	8 (15%)	6 (12%)	28 (19%)
Bad (patients, %)	6 (13%)	0 (0%)	0 (0%)	6 (4%)
Total (patients)	44	53	49	146

LWM LumiVision® water mixture, GBM gadolinium water mixture

L LumiVision® undiluted

^a^*p* value is 0.002 for GBM versus LWM in reader 1

^b^*p* value is 0.0008 for L versus LWM in reader 1

^c^*p* value is 0.003 for GBM versus LWM in reader 2

^d^*p* value is 0.0009 for L versus LWM in reader 2

The excellent rating for the contrast of the esophagus in readers 1 and 2 showed also a statistically significant difference between GBM and LWM (*p* = 0.002/*p* = 0.003), as well as between L and L WM (*p* = 0.0008/*p* = 0.0009). Between GBM and L, no statistically significant difference (*p *> 0.5) could be evaluated in both readers.

### Traceability of the bolus

All boluses could be followed well. The bolus tracing with GBM were rated to be excellent in 75% (40/53) and 77% (41/53) (R1 and R2) of the cases, but only in 50% (22/44) of the cases with LWM for both readers (Table 4). The traceability with L undiluted was rated to be excellent in 78% (38/49) and 70% (34/49) (R1 and R2) of the cases. There was a statistically significant difference of the excellent results between GBM and LWM (*p* = 0.02/*p* = 0.011), as well as between L and LWM (*p* = 0.0009/*p* = 0.008) in rating the image quality of readers 1 and 2. Between GBM and L, no statistically significant difference (*p* > 0.5) could be evaluated in both readers.

**Table 4:** Evaluation of traceability

Traceability	Contrast agents			
	LWM	GBM	L	Total
Reader 1				
Excellent (patients, %)	22 (50%)	40 (75%)^a^	38 (78%)^b^	100 (69%)
Fair (patients, %)	10 (23%)	10 (19%)	7 (14%)	27 (18%)
Bad (patients, %)	12 (27%)	3 (4%)	4 (8%)	19 (13%)
Reader 2				
Excellent (patients, %)	22 (50%)	41 (77%)^c^	34 (70%)^d^	97 (66%)
Fair (patients, %)	15 (34%)	10 (19%)	10 (20%)	35 (24%)
Bad (patients, %)	7 (16%)	2 (4%)	5 (10%)	14 (10%)
Total (patients)	44	53	49	146

LWM LumiVision® water mixture, GBM gadolinium water mixture

L LumiVision® undiluted

^a^*p* value is 0.02 for GBM versus LWM in reader 1

^b^*p* value is 0.0009 for L versus LWM in reader 1

^c^*p* value is 0.011 for GBM versus LWM in reader 2

^d^*p* value is 0.008 for L versus LWM in reader 2

### Interrater agreement

**Fig. 3:**
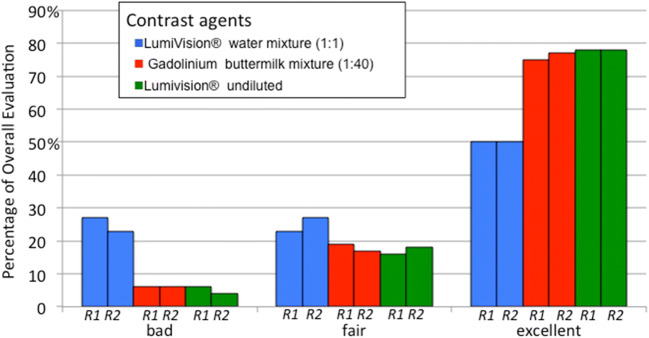
Overall ratings. The overall ratings were nearly equal in both readers with regard to the three different contrast agent groups

A substantial agreement could be reached with kappa = 0.661, 0.691, and 0.738 regarding the three different rating features (Fig. 3).

## Discussion

With the advantages of ultrashort sequences, MR swallowing has changed from a pure morphologic imaging technique to a technique, which combines morphology with functional imaging. The unique advantages of arbitrary slice angulation and the lack of ionizing radiation made MR fluoroscopy a valuable tool in imaging of GERD [3, 12] or postoperative failure after fundoplication [4–6]. The use of ultrafast imaging technique allows real-time imaging of swallowing and enables visualization of peristalsis and bolus transport of the esophagus [1, 13], which can add important information to the static morphologic assessment.

Multiple oral contrast agents have been used in esophageal MR imaging like pineapple juice, blueberry juice, water mixtures, and gadolinium chelate mixtures [2–6, 9, 12, 14]. However, it is still unclear which substances provide the best luminal contrast. No clear recommendations were given for dynamic imaging of swallowing in MRI to obtain good luminal distention, good contrast to surrounding structures, and good traceability of the swallowed bolus.

With regard to luminal contrast, distention, and traceability of the bolus, the contrast agent LumiVision® was evaluated undiluted and diluted with water, as well as a gadolinium-buttermilk mixture. The gadolinium-buttermilk mixture was previously found to be a good contrast medium, except in patients with lactose intolerance or milk allergy and those recommended to be given a dilution of 1:40 [15].

The ideal oral contrast medium should fulfil certain requirements for a safe and effective application. It should be easy to administer, rapidly eliminated, if necessary, of appropriate viscosity for administration, and should have a broad availability. Another important reason is the lack of side effects. Up to now, there is still a discussion of possible gadolinium contrast agent toxicity due to repeated oral ingestion of gadolinium and deposition in the body or brain with possible consequences [16]. So, if possible, a contrast agent, containing natural substances like fruit juices, as we ingest more or less daily, should be preferred, except the patient has fructose intolerance.

Pineapple juice was found to be the fruit juice with the highest signal intensity of the natural contrast agents due to the highest manganese content [17, 18], but commercially it is available in packs containing various dilutions of juice with various unpredictable manganese levels [19]. The same problem appears with blueberry juice, which is mostly not available undiluted, and so contains only a little manganese content. LumiVision® (b.e. imaging GmbH) [20] was commercially launched as a potential alternative with natural content to existing oral contrast agents in 2015, either for signal suppression of gastrointestinal tract in T2-w MRCP signal or to increase signal intensity on T1-w images. Therefore, it can also be used as an oral marker on MR swallowing examinations [4]. The biggest advantage of LumiVision® is that its preparation is controlled and always is produced with the same concentration and consistency.

This study presents the results of the commonly used gadolinium-buttermilk mixture compared to the gadolinium-free oral LumiVision® (undiluted or diluted) in MR fluoroscopy with regard to image quality. No statistically significant differences were found comparing the commonly used gadolinium-buttermilk mixture and undiluted LumiVision® in the present study regarding all examined image quality features in the overall evaluation, including the contrast between the esophageal lumen and the surrounding structures and esophageal distention as well as traceability of the bolus.

Undiluted LumiVision® was clearly superior in image quality to diluted LumiVision® regarding all features evaluated, especially considering the contrast between the lumen and surrounding soft tissues.

There was also superior evaluation of distention, contrast, and traceability between the GBM group and the LumiVision® diluted group. For the purpose of luminal contrast and bolus traceability, a “bright” esophageal filling is indispensable, and this can be fulfilled by using GBM or undiluted LumiVision®.

To the best of our knowledge, this paper presents the first experience with the use of undiluted LumiVision® for esophageal MR imaging. Our group was the first that demonstrated the feasibility of visualization of the esophagus with oral gadolinium-buttermilk mixture [3–5]. With the present MR protocol, we can achieve the same image quality with a gadolinium-free contrast agent. Even if orally administered gadolinium-based contrast agents were almost completely excreted in the feces and not absorbed [21], the use of a contrast agent like LumiVision® consisting of concentrated fruit juices seems to be a good way of reducing synthetic contrast media. It has been reported that failed esophageal visualization is related to delayed swallowing of a contrast agent or rapid passage of thin liquids through the esophagus [22]. Therefore, LumiVision® undiluted, which has a thick consistency like nectar, is ideal to evaluate the esophageal transit time and peristalsis. This might be one explanation why LumiVision® diluted reached inferior ratings. First of all, the consistency might be too thin, and the bolus transit cannot be followed very well. Secondly, dilution reduces the manganese content, and so the contrast and also the traceability were rated inferior.

This study has some limitations. First, this study might include a possible selection bias. Due to the retrospective study format, the choice of the contrast agent was based upon the time of examination and not on randomization. Second, the results were not evaluated with regard to the two different MR machines. A 3 T MRI usually reaches higher contrast due to signal-to-noise ratio and contrast-to-noise ratio. But it is known that especially artifacts of the balanced steady-state free precession sequences known from 1.5 T increase on 3T MRI due to B0 inhomogeneities; so often more susceptible and pulsation artifacts are observed with 3T MRI [23].

However, the examination procedures were assumed to be in line with radiologic standards, and on both MR machines, SSFP sequences with similar pulse sequences parameters regarding spatial and temporal resolution were used. Thirdly, we did not do a quantitative analysis in vitro with the different contrast agents, as well as a quantitative analysis of the contrast between the esophageal lumen and the surrounding structures.

All these limitations seem acceptable considering that this survey-based study showed high interrater correlations. Further prospective studies are essential to confirm our results.

In conclusion, LumiVision® undiluted is as hyperintense as the gadolinium-buttermilk mixture in MR fluoroscopy and shows a similar consistency. Therefore, undiluted LumiVision® may be preferred for safe esophagus visualization and motility evaluation in patients after Nissen fundoplication.

## Abbreviations

B-FFE: Balanced fast-field echo sequence
GERD: Gastroesophageal reflux disease
MRI: Magnetic resonance imaging
TrueFISP: True fast imaging with steady-state precession

## Declarations

### Guarantor

The scientific guarantor of this publication is Christiane Kulinna-Cosentini, MD.

### Statistics and biometry

Michael Weber kindly provided statistical advice for this manuscript. He has significant statistical expertise. No complex statistical methods were necessary for this paper.

### Study subjects or cohorts overlap

Some study subjects or cohorts have been previously reported in:

Swallowing MRI a reliable method for the evaluation of the postoperative gastroesophageal situs after Nissen fundoplication. Eur Radiol. 2019 Aug;29(8):4400-4407.

### Methodology

• retrospective

• case-control study

• performed at one institution
